# Attitudes of Polish Doctors towards Brain Death

**DOI:** 10.3390/ijerph192113729

**Published:** 2022-10-22

**Authors:** Krzysztof Leśniewski, Beata Antoszewska, Bożena Baczewska

**Affiliations:** 1 Department of Orthodox Theology, Faculty of Theology, The John Paul II Catholic University of Lublin, Al. Racławickie 14, 20-950 Lublin, Poland; 2 Department of Special Needs Pedagogy and Resocialisation, Faculty of Social Sciences, The University of Warmia and Mazury in Olsztyn, Żołnierska 14, 10-561 Olsztyn, Poland; 3 Department of Internal Medicine and Internal Medicine in Nursing, Faculty of Health Sciences, Medical University of Lublin, Chodźki 7, 20-093 Lublin, Poland

**Keywords:** death, brain death, neurological criteria of death, attitudes towards brain death

## Abstract

Brain death has been one of the most controversial issues in the medical and bioethical debate globally for more than fifty years. There is no unanimous acceptance of the understanding of brain death, and a single set of neurological criteria for the determination of human death that is accepted worldwide has yet to be established. Physicians from different specialisations understand brain death differently. Therefore, research is needed to understand and typologically classify their points of views as regards brain death. In Poland, this research is particularly important, as the views of anaesthesiologists, neurologists and transplantologists, who fully accept and support brain death as being synonymous with biological human death, have dominated the scholarly debate on this issue. This study presents the opinions of Polish physicians with various medical specialisations in relation to brain death. Free-form interviews with 28 doctors were conducted. Participants expressed their personal views on brain death, while exhibiting at the same time various emotions. We discuss our findings in relation to the existing framework of knowledge and debate concerning brain death and the Polish legal regulation in force when the interviews were carried out. Although participants had different beliefs with regard to brain death, the research team managed to classify their statements and opinions into five attitudes, taking into account what for them were the most important, namely: the escapist–protective attitude, the scientistic–medical attitude, the accepting–critical attitude, the ignorant–agnostic attitude, and the ambiguous attitude.

## 1. Introduction

Death is one of the most perplexing issues, and has been the subject of reflection among thinkers, experts in various fields of science, and ordinary people since time immemorial. Overall, it can be said that reflection on death entails consideration of philosophical, bioethical, theological, medical, legal and social grounds. However, it is worth noting that even in ancient times, philosophers thought of death in different ways. The notion of death as the separation of soul from body goes back to Plato (Phaedo 64c, 67d) [1,2]. In contrast, according to Aristotle, life is maintenance of the vital heat and death is its destruction (De Iuventute et Senectute 469b, 19–20). The cause of death is, from this perspective, the failure of the organ that contains the source of the essential nature, which is the heart in humans (De Iuventute et Senectute 478b 32–33) [3]. Still, for time immemorial, it has generally been accepted that death occurs when the heartbeat and breathing stop. Yet, on analysis of the literature on the subject, it is difficult to determine precisely when, why and by whom the criteria of cessation of blood circulation and heartbeat was debated and accepted. In its essence, death is not only the end of a person’s biological life, but also an ontological event and a mystery incomprehensible to human rationality.

It can be said, however, that the continuity of the cardiovascular function has always been treated as a critical feature of life. This classical understanding of death became insufficient in the late 1960s, when the Harvard Committee proposed a new definition and new criteria for determining the death of an individual [4,5,6]. The new criteria for determination of death, referred to as neurological ones, did not refer directly to the cessation of cardiovascular and respiratory functions but relied on diagnosing permanent and irreversible cessation of the brain and brainstem function and permanent loss of consciousness [7].

This new neurological concept of death has sparked endless debates among scholars, who are constantly raising arguments both for and against the legitimacy of brain death [8,9]. The medical community is particularly divided on this issue [10,11,12]. Some medics claim that a person ceases to exist as a human being when his or her consciousness ends—even though some biological functions of the body are sustained, the person should, in their view, be considered dead. However, others believe that death occurs only after the vital functions of the body cease and certain characteristic signs of death, such as rigor mortis or livor mortis, appear. Doctors who criticise the neurological definition of death stress that it is difficult to consider bodies of patients with evident signs of life as dead; they also raise the issue of the continuous evolution of neurological criteria for the determination of death and the absence of clarification of either the said criteria or basic and ancillary tests that would be accepted globally [13,14].

For over 60 years now, biological justification of the concept of brain death has prevailed, even though medical observations imply that bodies of patients in this condition can demonstrate various integrative functions even after a long time. Thus, the assertion made by some neurologists and anaesthesiologists that the death of the whole body will inevitably take place soon after brain death has been diagnosed is debatable [15]. This should also make us pause to ponder whether the confirmation of a permanent and irreversible loss of all brain functions (in the case of Poland, since December 2019, it is performed by two consultant doctors) should be treated as an empirical fact or if it is merely a formal decision that makes it possible to harvest organs from the so-called dead donor. Thus, a question arises: how do consultants perceive a person’s death? Is death the loss of one’s personal identity, or is it the complete loss of the human organism’s somatic integration [13], which is manifested by the cessation of circulation and respiration? Can a human body be considered dead when it becomes biologically incapable of manifesting conscious (sensory or rational) functions as a result of some brain damage?

Asking these questions opens the proverbial Pandora’s box that unleashes numerous biological, bioethical, theological, social, and legal problems to contend with [16]. The search for answers to the questions concerning the understanding of brain death is therefore an immensely difficult task. It is not a purely theoretical issue, but also an existential problem, which is particularly challenging to surgeons and physicians, not only those who are directly involved in the determination of brain death or organ transplantation but also representatives of other specialisations who feel obliged to reflect on the threshold between life and death. The lack of unanimity among doctors regarding the legitimacy of the concept of brain death and the criteria applied to its determination raises concerns, which inclines us to enter into a dialogue with doctors on this subject.

In the modern world, we observe growing tendencies to replace the Hippocratic tradition with a new system of medicine, the essence of which would be to ration medical care by seeking a compromise between utility and equality. The adherence to the Hippocratic ethics is seen as too expensive in some societies since it obliges doctors to help every patient within the limits of their abilities and discernment, regardless of the diagnosis of the patients’ state of health. When reforming a country’s medical care system, the priorities established by institutions that distribute healthcare funds increasingly often take precedence over patients’ priorities—especially the priorities of patients who are predicted to soon pass away or experience a low quality of life [17].

Some consultants are not fully convinced that death has occurred when it is determined based on neurological criteria, which presumably affects their rapport with patients. Hence, gaining insight into the brain death-related beliefs of doctors representing different medical specialisations can help evaluate the relationship that such doctors have with patients in the case of whom it is difficult to unequivocally determine whether they are still alive or already dead. The ethical and medical attitudes of doctors to this special group of patients, i.e., patients who are confirmed to be brain dead, are significant. Thus, far, the medical and legal regulations pertaining to brain death and neurological criteria for determination thereof have not been specified in so precise and unchangeable a manner as to ensure absolute certainty in declaring an individual’s death. These medical and legal norms are constantly evolving, and efforts are made by medical authorities to formulate such norms so that they will be acceptable in all countries across the world.

The main goal of the article was to specify the types of attitudes of medical specialists in relation to human death, which has been the subject of a debate on the criteria for its determination, which has been going on for several decades. The qualitative studies carried out allow us to gain a deeper insight and to evaluate the doctors’ attitudes toward patients who are nearing death. The way patients are treated by doctors depends on how the latter understand what a person’s death is and what its signs are. By gaining knowledge about doctors’ attitudes towards brain death, we should be able to identify more precisely the problems that medics struggle with and contribute to the improved quality of medical and non-medical care for patients in the agonal phase.

## 2. Materials and Methods

In 2019, in Poland, qualitative studies were conducted in order to learn the opinions of doctors of various specialisations as to their understanding of brain death and to create a typological qualification of their views. The research team conducted twenty-eight free-form interviews with Polish consultant doctors from different medical centres (across Poland) and analysed the results to identify their understanding of brain death. It should be mentioned that each interviewed consultant had been informed about the problem area and the aim of the interview, as well as the role of the interviewer, and gave informed consent to participate in the study.

The objective of our study was to learn the opinions of specialist doctors on the criteria of determining brain death, including their personal experiences as regards the death and dying of patients, in particular in cases of brain death. The subject of this study was defined as the opinions of doctors toward the death of patients, especially toward brain death. The motivation was the lack of consensus in scientific discourse as regards the definitions of brain death and the determination of brain death criteria. This research objective can be briefly expressed by the following question: How do doctors of different medical specialisations understand brain death, and how to define their attitudes towards it?

The questions for interviews were prepared at the stage of defining the research aim and reviewing the issues of death and brain death. These instructions were then modified during the study to best adjust them to the course of every interview with a specific interlocutor, while maintaining the focus on the methodological framework that had been prepared beforehand. In our qualitative research, as a research method, we used the interview with prepared dispositions, the order of which was modified. We decided that it would be appropriate, in tune with Steinar Kvale, that “Simply expressed, the more spontaneous the interview procedure, the more likely one is to obtain spontaneous, lively, and unexpected answers from the interviewees” [18].

The research material obtained from the interviews was then read through several times to determine the concept categories for which such material was analysed. Next, the material referring to the research problem was extracted from the responses given by the doctors during the interviews, and the most frequent topics were identified. The interviewees are referred to in this article as interlocutors or narrators. The voices of the narrators are highlighted in the article by quoting the most important excerpts from the interviews. This is reflected in the relatively large number of quotations, which are an attempt to reconstruct the participants’ viewpoints and to illustrate the fact that different narrators may have similar reflections on a given subject.

A target manner of selection of interviewees for this study was applied. Ethical standards were observed during the interviews, including maintaining personal data confidentiality. It was also ensured that the research material obtained would not be made accessible to third parties and that the research material analysis would only be used for scientific purposes. The medical consultants who were interviewed agreed to take part in the interviews and to have them recorded, as well as to the publishing of certain fragments of their statements that would prove significant during the analysis.

The selection of the group of interviewed doctors was deliberate, taking into account various cases (differences in age, sex, seniority, professional experience and medical specialty). Inclusion criteria for the study were: no relationship with the interviewer, and free consent to participate in the study.

The interviews with specialist doctors were carried out in different parts of Poland between March and December 2019. The interview location was chosen individually by each doctor. The interviews lasted from 45 to 90 min. The main part of each meeting was the interview proper, which was recorded on a dictation machine, and then transcribed. A total of 28 in-depth individual interviews were conducted with both male and female specialist doctors of different ages, and therefore, different seniority, who were working in various types of medical institutions. Table 1 below contains basic information on the interlocutors, arranged according to the order in which the interviews were carried out.

Of those who participated in the study, there were 15 females (53.57%) and 13 males (46.43%). The average age of the interviewees was 57.39 ± 9.01 years, while the average seniority was 31.82 ± 9.13 years. The study participants had various specialisations, the most numerous were oncologists (*n* = 7, 25.0%), anaesthetists (*n* = 6, 21.43%) and neurologists (*n* = 4, 14.29%), and among oncologists, one doctor had, additionally, a specialty in gynaecology. The characteristics of interviewees are presented in Table 2.

All interviews began with a casual conversation. The narrators were informed about the purpose of the study and the way the information they would provide would be used. It is worth emphasising that the interlocutors differed in their commitment to answering the questions and sharing their experiences related to the death of patients. It should be noted that doctors spoke about brain death in both emotional and reflective ways. They struggled with their thoughts; this was evident in terms of started and unfinished sentences, repeated phrases, changing the subject, raising or lowering the tone of their voice, prolonged silences, avoiding eye contact, and expressive nonverbal sounds. Their range of statements varied from decisiveness to doubts, restraint and an absence of an unambiguous position on the subject of brain death. We selected the most important statements from the extensive research material. The statements cited below did not undergo any modifications, and have been cited exactly as expressed. Two independent reviewers categorised the transcribed interviews, ensuring that intercoder reliability was gained.

### Statistical Analysis

Counts and percentages were used to characterise the study participants. Moreover, descriptive statistics, such as mean, standard deviation, median, minimum, and maximum, were applied. The Fisher’s exact test and the ANOVA test were used to check whether doctors’ attitudes towards brain death differed by age, seniority, gender, and specialty. The assumptions of the ANOVA test were met: 1. the distribution of age and seniority in the analysed groups was similar to normal as revealed by the Shapiro–Wilk test; 2. the variances in the groups were shown to not differ significantly by Levene’s test. Statistical analysis was performed using IBM SPSS Statistics, version 28.

## 3. Results

As a result of the analysis of the material obtained from the interviews, the researchers defined five basic attitudes of doctors toward brain death: the escapist–protective attitude, the scientistic–medical attitude, the accepting–critical attitude, the ignorant–agnostic attitude, and the ambiguous attitude.

### 3.1. The Escapist–Protective Attitude

The first attitude of doctors in relation to brain death was defined as the escapist–protective attitude (represented by ten interlocutors: 1, F; 3, F; 6, F; 7, M; 8, F; 10, F; 15, F; 18, M; 19, F; 23, F). This group of interviewees was the most cautious when talking about brain death, and sometimes even tried to avoid answering the question about the neurological criteria of death. They were self-constrained when speaking about brain death. Their narratives were characterised by longer intervals, hesitation, and the search for the right words to express their opinions. The interlocutors undoubtedly tried to avoid answering this question:

They are very different types of death, I mean brain death looks different, cardiological death looks different, and metabolic death may look different, in brief: a cascade of things that are occurring at the time.

(7, M)

The interlocutors justified distancing themselves from the issue by having insufficient knowledge:

I may not know enough about this subject.

(1, F)

The criteria were adopted, and I don’t know entirely if it is really so, if … if …

(18, M)

I don’t have to declare brain death… so I don’t have to adapt to these contemporary definitions; in my opinion, the circulatory and respiratory criteria are the most proper: cessation of breathing, cessation of the heartbeat, decreasing body temperature, and after some time, livor mortis.

(8, F)

What is characteristic of this attitude is that the interlocutors emphasise their lack of competencies in connection with brain death:

I mean, I don’t understand it, so let’s not discuss it, it’s too complicated for... I don’t comprehend this definition

(3, F)

The interlocutors underlined that they were in a more comfortable situation because they did not have to declare the brain death of patients:

I am in a rather good situation because I do not have to respond to this on a daily basis, and honestly, it is such a difficult issue that I’m not even sure of my answer because, as I say, there’s a very thin line between life and death, which is very easy to cross, and I’m always worried that these debates will actually lead to a growing consent to euthanasia.

(15, F)

Besides the unequivocal avoidance of the subject of brain death, the statements also show that the interlocutors phrased their replies in a way that would not conflict with the Announcement of the Minister of Health of 17 July 2007 on the criteria and method of determining the permanent irreversible cessation of brain function [19].

It is always a difficult question, but for me, these are purely theoretical things because I… I do not work in this field, so… it is always a dilemma. Yes, there’s the question if it is the death of the brain that occurs 4 min after the blood circulation stops, but… is it complete death when all organs stop… This is a slippery subject, so very imprecise; I think, that there is no good definition here … the cardiorespiratory criteria are one hundred per cent certain.

(19, F)

The essence of the escapist–protective attitude lies in both the uncertainty and difficulty in taking a stand on brain death and the binding neurological criteria of its determination. When asked detailed questions, the specialist doctors in this group referred to the aforementioned Announcement, while admitting that this was not a subject of their deeper interest. This attitude seems more characteristic of women than men. A possible reason is that many doctors who share this attitude practise a medical speciality that does not directly touch on the problem of brain death.

### 3.2. The Scientistic–Medical Attitude

Some opinions on the neurological criteria of death were expressed in a way that could be classified as the scientistic–medical attitude (eight interlocutors: 2, M; 4, F; 14, M; 16, M; 20, M; 24, M; 25, F; 26, M). This attitude is characterised by the lack of any doubts about whether a medical error is possible when the currently binding brain death criteria in legal regulations are applied. The following narratives are examples of this way of thinking:

The death of a patient occurs when the brain dies. We consider brain death to be death, as it is generally known, so until brain death has not been determined, especially determined twice, we cannot take organs for transplantation, so it shows how this problem can be viewed, so in the field of transplantology, we can read that the death of a person is the death of the brain.

(4, F)

The progress in medicine guarantees the certainty of a diagnosis. We have this kind of research available; it seems, in my opinion, these neurological criteria are very certain. I don’t think there could be any shadow of a doubt here.

(26, M)

There are the most important groups of cells in the brainstem that determine whether a person is alive or dead, and these are the centres that control respiration, right, and the entire course of the examination by the commission is to demonstrate whether these structures are alive or dead, and if they are dead it means that this person is dead but some of his or her cells are alive; we cannot really say that a whole man dies at once, otherwise we could not take organs for transplantation. This matter here is quite unambiguous: irreversible cessation of circulation or the death of the brain with the brainstem, where the patient will not breathe are the death criteria adopted worldwide and one must accept it, and questioning it is … seems to be irrational or arises from ignorance or the lack of proper discipline of thought.

(20, M)

In my opinion, death occurs when the brain stops working, when the work of the organs is no longer being organised, and although the heart still works for some time… although it appears that some organs or parts of the body are functioning. Well, when the patient meets all the criteria of being dead in the neurological diagnostics, we consider this patient to be dead and terminate the treatment; we conclude that he is dead.

(14, M)

In the case of brain death, everything is evident because we can have a situation when the brain is dead, but all other organs work well: the kidneys and liver, the pancreas, the lungs, the heart they all work; but despite this, I think this is already the death of the body. This is the death of the body as a whole, though single organs can still work… this is obviously very difficult psychologically.

(24, M)

It follows from the above statements that the doctors accepted the provisions of the Announcement, considering them to be certain and commonly binding. This document, in their opinion, is understandable and clear. It provides the essential and indisputable knowledge in a systemised manner, making it possible to determine brain death based on scientifically proven criteria with no risk of making a mistake. They were convinced that the Announcement provided specialist doctors and consultants with specific diagnostic tools that could guarantee they would not err. In this approach, the interviewees underlined the trust they had in the legally determined criteria and their role in the development of medicine and in saving human lives. Some narrators did not reflect deeper on the neurological criteria for the determination of a person’s death or the concept of brain death as such. They firmly believed in the correctness and legitimacy of the legal and medical regulations contained in the Announcement that was then in force. The interlocutors also emphasised the constant progress in medicine in the field of examinations employed to determine brain death. It is worth noting that while the specialist doctors pointed out that all possibilities of saving a patient must be exhausted and the brain death diagnosis must be certain, they were aware that this creates a mental burden for any anaesthesiologist involved. Most interviewees in this group underlined the precision of the legal and medical contents of the Announcement and the legitimacy of requesting team decisions in the determination of brain death. The specialist doctors representing the scientistic–medical approach considered it completely pointless and unjustifiable to initiate any debates on brain death and rejected any possibility of questioning the neurological death criteria.

### 3.3. The Accepting–Critical Attitude

The third characteristic approach of specialist doctors towards brain death is the accepting–critical attitude (represented by seven interlocutors: 9, M; 12, M; 13, M; 17, F; 21, F; 22, F; 27, M) concerning the provisions of the Announcement of the Minister of Health of 17 July 2007 on the criteria and method of determining the permanent irreversible cessation of brain function. When referring to the above document, some narrators discussed certain fragments and reflected upon them or analysed them critically:

Well, I think that it is not always such an evident case, that may not, but… well, of the tests we use here, we do not make cerebral examinations, but we do it based on… well, cessation of heart function, respiration. I have doubts… I have serious doubts–once I start pondering on it. Especially that these… well, not exactly numerous cases, do occur anyway…

(13, M)

I persistently return to this point that for me, death is the cessation of spontaneous heartbeat and respiration. Of course, someone might say: all right, but there are the centres… below there… exactly… which control the mechanisms of respiration and cardiac function by sending impulses, well, this is true; however, the situation is usually reverse, that is, the cessation of the cardiac function and respiration leads to the death of the brain due to the spontaneous cessation of the heartbeat and respiration. To me, this means death!

(9, M)

I know it is determined here, right, the death of the entire brain’s brainstem, because one can rely on imaging examinations, dynamic imaging, right, but there may not be such entirely unambiguous symptoms. We do not know completely what is the moment, despite all those presumably accepted circulatory-neurological criteria. I think that as long as we deal with a patient in a vegetative state, and we have examples of people who wake up after years of being in such a condition I would not undertake… to determine brain death. Yes, yes… although I know I have many opponents in this regard.

(27, M)

In our daily medical practice… you can’t deliberate, ponder on every patient. We need to decide: is he alive or not alive. If we as doctors stood over everyone wondering if they are still alive, but maybe not entirely alive, or perhaps they are a little alive or maybe not, well then… well, it can’t be like this, we need to adopt some criteria. I don’t know [clearing the throat]; it is difficult for me to say but there must be ongoing research of this sort.

(12, M)

Despite referring to the Announcement—the law in force at that time—this group of specialist doctors noticed the inconsistency of the provisions contained in it. They shared their doubts concerning the subject content and the formal solutions. They were convinced that doctors who diagnosed brain death according to the neurological criteria experienced various moral and ethical dilemmas. Referring to their medical knowledge, the interlocutors hoped that the criteria for determining brain death would ultimately be made more precise, so that the basic and auxiliary examinations would ensure unquestionable certainty and prevent any medical errors. They also underlined that their doubts concerning the current neurological criteria and procedures for the determination of brain death were justifiable. They cited cases of patients diagnosed with brain death who regained consciousness, which were reported in the subject literature and mass media. They also recalled cases of patients who had severe brain injuries but recovered after rehabilitation. They wonder how it was possible for a woman determined to be brain dead to give birth to a healthy baby [20,21]. They also asked why there were no uniform neurological criteria for the determination of death that would be implemented in all countries across the world. It is worth underlining that the consultants who represented this approach were characterised by being reflective and open to sharing doubts that this problem gives rise to, be they medical, legal, ethical or moral ones. They expressed their opinions with certain reserve because their experience told them that such opinions were unacceptable to some in the medical community, which could result in ostracism.

### 3.4. The Ignorant–Agnostic Attitude

The analysis of the narratives obtained proved that a lack of interest in the question of brain death among medics is also possible. This approach can be termed the ignorant–agnostic attitude (represented by two interlocutors: 11, F; 28, M).

I don’t remember it clearly… Well, if it is assumed that transplants and taking organs from donors are, shall I say, acceptable and good, then there must be such criteria.

(28, M)

Well, let me put it like this: I have mixed feelings, the neurological criteria were presented to us at some stage of my medical studies, I believe it was in the course of anaesthesiology in the fifth year, but roughly, briefly, and I have never explored this deeply afterwards.

(11, F)

This approach shows the indifference of specialist doctors to the problem of death. The representatives of this attitude (an oncologist and an anaesthetist) have direct contact with dying patients. The interlocutors tried to justify their viewpoints toward death by the fact that the patients they treated did not belong to the group of potential organ donors, and therefore, they simply did not need the knowledge they had once gained about neurological criteria of death.

### 3.5. The Ambiguous Attitude

There was only one interlocutor who expressed her attitude toward brain death in a way that can be classified as the ambiguous attitude (5, F). This is evidenced by the following excerpt from her narration:

There are no neurological criteria for death. What does ‘neurological’ mean? That this person is unconscious? Or that he is in a vegetative state? He is alive! He is warm, there is no body stiffness! He is warm, soft… The circulation is functioning, the respiration is functioning, and therefore, we do not entirely know, we are unable to state with complete certainty that the brain is dead. There are examinations which confirm with absolute certainty the death of the brain and then organs can be transplanted. Unless we have this certainty… You know, I am the secretary of the Ethics Committee of the University Hospital and the secretary of the Ethics Committee of the District Medical Chamber as well. So, we have this problem… these various ethical problems because there are such… There is no such thing as neurological death.

(5, F)

Some contradictions are noticeable in this narrative. On the one hand, the interlocutor negated the neurological criteria of the death of a person, but on the other, she considered it justifiable to conduct tests whose aim is to determine brain death. While emphasising the value of human life and the necessity to save a patient in a situation when there are still signs indicative of the patient’s physiological processes, she also refers to the certainty seemingly provided by the examinations intended to diagnose brain death.

### 3.6. Overview of the Attitudes towards Brain Death

**Figure 1:**
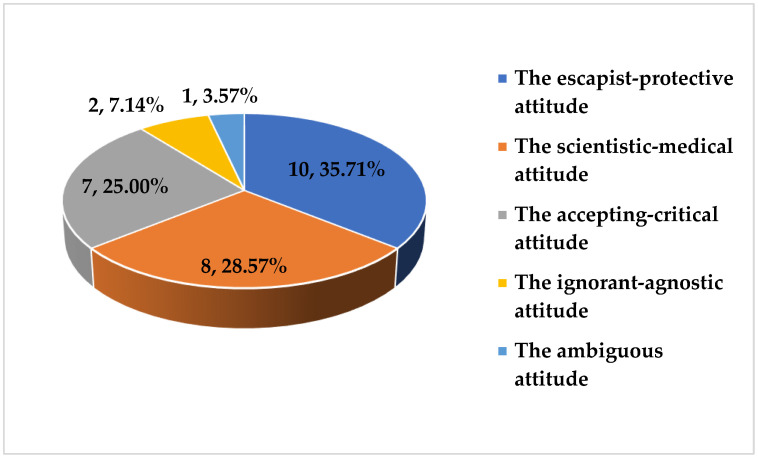
Attitudes of the interviewees towards brain death.

In conclusion, the escapist–protective attitude, the scientific-medical attitude and the accepting–critical attitude were the most common among the specialist doctors participating in the study. Moreover, interviewees displayed the ignorant–agnostic attitude or the ambiguous attitude definitely less often (Figure 1).

In our study, we further compared the attitudes of specialist doctors towards brain death depending on age, seniority, sex and specialty. The ignorant–agnostic attitude and the ambiguous attitude were excluded from the analysis due to the small number of cases. It turned out that the respondents with different attitudes towards brain death did not differ significantly in age (*p* = 0.723) or seniority (*p* = 0.592). However, females presented the escapist–protective attitude more often, while the scientistic–medical attitude was more common in males, but the result of Fisher’s exact test was not statistically significant (*p* = 0.066). There were also no significant differences in the attitude towards brain death depending on the specialty (*p* = 0.388). However, it should be noted that the sample size was small, and this requires further research. The results of the above analyses are presented in Table 3.

## 4. Discussion

The problems raised in this article correspond to the need to expand the scope of scientific research dealing with the borderline between human life and death. The research conducted so far shows that “though most clinicians know and personally endorse the legal criteria for declaring death, some may still feel uncomfortable with the concept of death and hesitant with the idea that brain death really equates to death” [22]. It must be stressed that even some neurologists do not have a consistent rationale for accepting brain death as death [23]. An in-depth bioethical reflection on the borderline between life and death based on qualitative studies creates a chance to gain new knowledge about this problem and to solve significant social issues which mankind is currently struggling with. Having a more precise definition of the borderline between life and death is crucial for every human being, regardless of his or her stage of life. The right to life is guaranteed by the Universal Declaration of Human Rights, which was adopted by the United Nations on 10 December 1948. Article 3 of this globally recognised document states that: ‘Everyone has the right to life, liberty and security of person’, while Article 5 says that ‘No one shall be subjected to torture or to cruel, inhuman or degrading treatment or punishment’ [24]. Since all human beings have immeasurable value, they must be respected, and the protection of their civil, political, economic, social and cultural rights must be ensured. This respect for every human being, guaranteed on the global level, also entails the right to medical care until the end of one’s life. The neurological criteria of human death and the concept of brain death introduced in 1968 continue to be a crucial problem for some members of the medical community. Despite the binding legal and medical regulations, specialist doctors in many countries across the world, including Poland, present different attitudes to equating brain death with a person’s death. Conducting interviews with doctors of different medical specialisations and their subsequent analysis provides vital research material that helps to determine the typology of doctors’ approach toward the concept of brain death, and thereby demonstrates the need for a more precise definition of the borderline between human life and death. By learning about the perception of brain death among doctors of different specialisations—and not only those who are directly engaged in determining brain death (in the case of Poland, anaesthetists and neurologists) or those who perform organ transplants—it is possible to carry out a more thorough analysis of this significant social problem. There is thus a need for further developing a comprehensive description of the understandings of doctors and other healthcare professionals regarding the meaning and determination of death [25].

The debate concerning brain death that has been going on for a few decades now is not purely a medical problem, but also an important bioethical, moral, social and legal problem [26,27,28,29]. The opinions given by the specialist doctors indicate that brain death may not invariably be tantamount to death [30].

The objective of this study was to learn the opinions of Polish doctors on the criteria for pronouncing patients brain dead, as well as their personal experiences regarding the death and dying of patients, and their opinions towards brain death. This was an exploratory study performed in an attempt to uncover a certain fragment of social reality. The way one reflects upon death is significant, because it affects the approach to life—not only one’s own, but also that of other people.

The doctors’ narratives analysed in this article lead to the conclusion that the medical community is divided, and not all of its members advocate in favour of the neurological criteria and definition of brain death contained in the Announcement of the Ministry of Health of 2007 [19]. The interlocutors present an array of attitudes towards the notion of brain death. Based on the analysis of doctors’ statements on brain death, five characteristic attitudes were distinguished: escapist–protective, scientistic–medical, accepting–critical, ignorant–agnostic and ambiguous. The majority of our interlocutors represented the escapist–protective approach, which was characterised by some uncertainty and avoidance of expressing their views on the neurological criteria of death or brain death. The specialist doctors who exhibited the scientistic–medical approach considered the medical and legal regulations contained in the Announcement of the Ministry of Health of 2007 to be indisputably certain. Therefore, many of them rejected the purposefulness or legitimacy of any scientific or social discourse on this subject. These doctors, whose attitude can be classified as accepting–critical, were highly cautious when evaluating the binding medical procedures regarding brain death. Referring to their specialist medical knowledge and experience, they raised some doubts in terms of the subject itself and the formal solutions; at the same time, they pointed to certain inconsistencies in the provisions of the Announcement. This group of medics were hopeful that the criteria for the determination of brain death would ultimately be made more precise and unequivocal. The other two approaches, that is, the ignorant–agnostic and the ambiguous, were characterised by indifference and the lack of need to have any knowledge about brain death.

## 5. Conclusions

The interviews conducted with Polish consultant doctors revealed other significant research areas that could help us gain a much deeper insight into the analysed issues related to brain death. There is an increasingly noticeable need to expand the research by including the views of other specialist doctors, particularly those who are directly involved in diagnosing brain death, i.e., anaesthetists and neurologists, as well as specialist doctors who perform organ transplants, that is, transplantologists. Interviewing doctors of these specialisations could provide highly pertinent research material, including their personal experiences of relationships with patients suffering brain death. It is crucial to gain knowledge of the views of doctors engaged in saving the life and limb of patients with severe disabilities (both organ donors and recipients) with a view to developing and improving the quality of medical and non-medical care.

## Figures and Tables

**Table 1:** Information on the interviewees.

Interview No	Interviewee Symbol	Age	Sex	Seniority	Specialty
1	1, F	60	Female	31	neurologist
2	2, M	65	Male	39	vascular surgeon
3	3, F	62	Female	37	oncologist
4	4, F	55	Female	29	transplantologist, nephrologist, internist
5	5, F	64	Female	38	anaesthetist
6	6, F	51	Female	25	internist, palliative medicine specialist
7	7, M	65	Male	39	paediatrician, palliative medicine specialist
8	8, F	60	Female	35	neurologist
9	9, M	55	Male	35	oncologist
10	10, F	58	Female	32	oncologist
11	11, F	35	Female	10	oncologist
12	12, M	62	Male	36	cardiac surgeon
13	13, M	57	Male	27	gynaecologist, oncologist
14	14, M	67	Male	37	neurosurgeon
15	15, F	45	Female	20	radiotherapist
16	16, M	52	Male	32	oncologist
17	17, F	76	Female	53	neurologist
18	18, M	60	Male	35	forensic medicine specialist
19	19, F	45	Female	20	family medicine specialist
20	20, M	59	Male	33	transplantologist
21	21, F	57	Female	32	neurologist
22	22, F	43	Female	16	anaesthetist
23	23, F	58	Female	27	anaesthetist
24	24, M	44	Male	19	anaesthetist
25	25, F	58	Female	37	cardiologist
26	26, M	57	Male	31	anaesthetist
27	27, M	67	Male	41	oncologist
28	28, M	70	Male	45	anaesthetist

**Table 2:** Characteristics of the interviewees.

Variables		M ± SD	Me (Min; Max)
Age		57.39 ± 9.01	58.0 (35.0; 76.0)
Seniority		31.82 ± 9.13	32.50 (10.0; 53.0)
		N	%
Sex	Female	15	53.57
	Male	13	46.43
Specialty	anaesthetist	6	21.43
	oncologist	6	21.43
	neurologist	4	14.29
	cardiac surgeon	1	3.57
	cardiologist	1	3.57
	family medicine specialist	1	3.57
	forensic medicine specialist	1	3.57
	gynaecologist, oncologist	1	3.57
	internist, palliative medicine specialist	1	3.57
	neurosurgeon	1	3.57
	paediatrician, palliative medicine specialist	1	3.57
	radiotherapist	1	3.57
	transplantologist	1	3.57
	transplantologist, nephrologist, internist	1	3.57
	vascular surgeon	1	3.57

**Table 3:** Attitudes of the interviewees towards brain death depending on age, seniority, sex and specialty.

Variables		The Escapist–Protective Attitude	The Scientistic–Medical Attitude	The Accepting–Critical Attitude	Statistical Test Result
Age		56.40 ± 6.98	57.13 ± 7.24	59.57 ± 10.33	F = 0.329, *p* = 0.723 *
Seniority		30.10 ± 6.82	32.13 ± 6.31	34.29 ± 11.48	F = 0.537, *p* = 0.592 *
Sex	Female	8 (61.54%)	2 (15.38%)	3 (23.08%)	*p* = 0.066 **
	Male	2 (16.67%)	6 (50.0%)	4 (33.33%)	
Specialty	anaesthetist	1 (25.0%)	2 (50.0%)	1 (25.0%)	*p* = 0.388 **
	oncologist	2 (33.33%)	1 (16,67%)	3 (50,0%)	
	neurologist	2 (50.0%)	0 (0.0%)	2 (50.0%)	
	other	5 (45.45%)	5 (45.45%)	1 (9.09%)	

* ANOVA test; ** Fisher’s exact test.

## Data Availability

The data presented in this study are available on request from the corresponding author.
